# Supplementary material for: α-Parvin promotes glucose uptake and metabolism in skeletal muscle with minimal influence on hepatic insulin sensitivity
Source: Mol Metab. 2026 Jan 21;105:102322. doi: 10.1016/j.molmet.2026.102322 (PMC12887382; doi:10.1016/j.molmet.2026.102322)
Supplement: Multimedia component 1 [file mmc1.docx]

**Supplemental Material**

**α-Parvin Promotes Glucose Uptake and Metabolism in Skeletal Muscle with Minimal Influence on Hepatic Insulin Sensitivity**

*Fabian Bock^1,2,3^, *Xinyu Dong^1^, Kakali Ghoshal^1^, David A. Cappel^4^, John W. Deaver^4^, Dan S. Lark^5^, Luciano Cozzani^4^, Deanna P Bracy^4^, Louise Lantier^4,6^, Allison Do^4^, Richard L Printz^4^, Santosh Thapa^7^, Owen P. McGuinness^4,6^, David H. Wasserman^4,6^, Ambra Pozzi^1,2,4^, Roy Zent^1,2,3^, Nathan C. Winn^4,7^

^1^Division of Nephrology and Hypertension, Department of Medicine, Vanderbilt University Medical Center, Nashville, Tennessee, USA

^2^Department of Veterans Affairs Hospital, Tennessee Valley Healthcare System, Nashville, Tennessee, USA

^3^Department of Cell and Developmental Biology, Vanderbilt University School of Medicine, Nashville, Tennessee, USA

^4^Department of Molecular Physiology and Biophysics, Vanderbilt University School of Medicine, Nashville, Tennessee, USA

^5^Department of Health and Exercise Science, College of Health and Human Sciences, Colorado State University, Fort Collins, CO, USA.

^6^Vanderbilt Mouse Metabolic Phenotyping Center, Nashville, Tennessee, USA

^7^Division of Gastroenterology, Hepatology, and Nutrition, Department of Medicine, Vanderbilt University Medical Center, Nashville, Tennessee, USA

*Co-first author

**
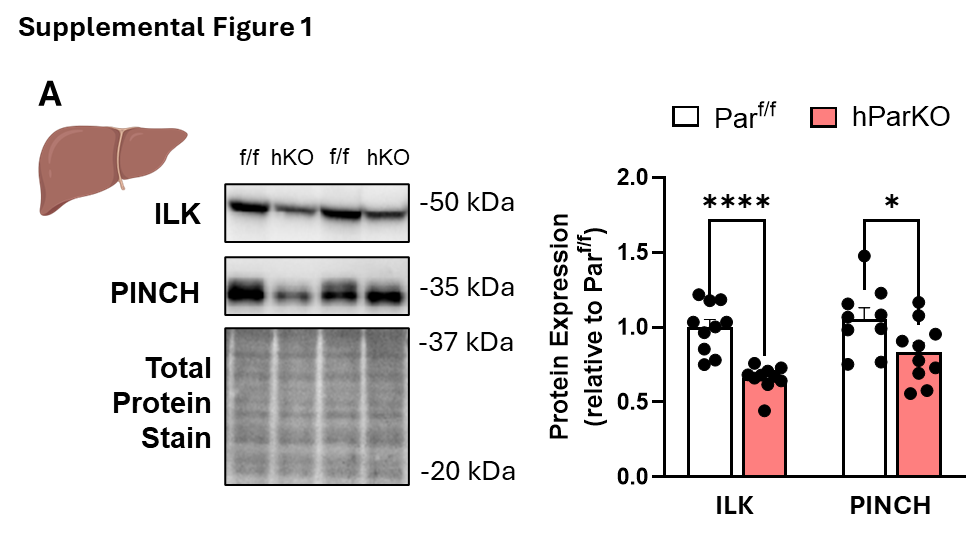
**

**Supplemental Figure 1** – Deletion of alpha-Parvin in hepatocytes results in a decrease in ILK and PINCH content. A) Immunoblotting was performed on liver lysates from mice with hepatic specific deletion of alpha-Parvin versus littermate controls. Independent samples T tests were conducted to test differences between groups. Data are presented as mean ± SE. n=9-10/genotype.

**
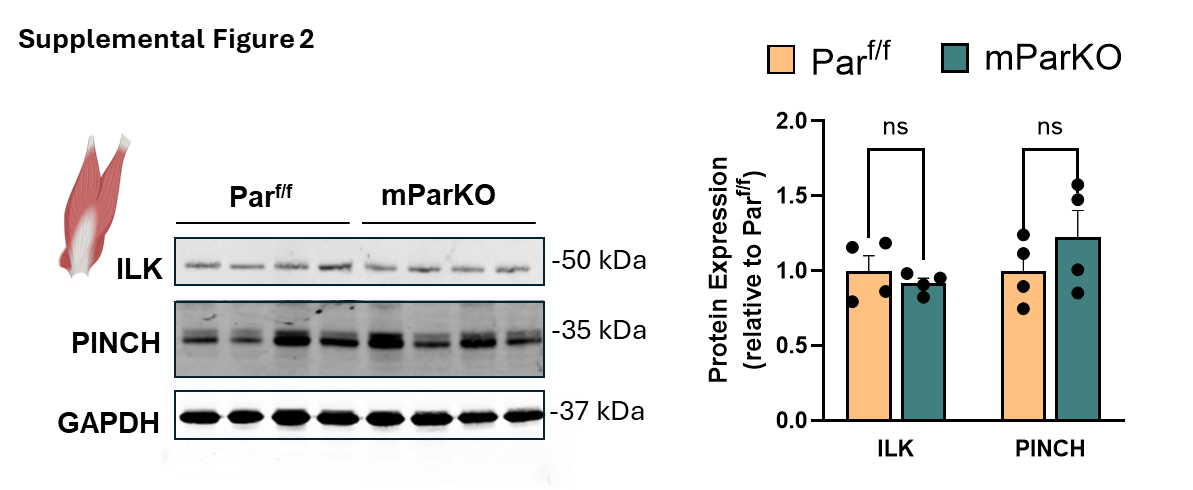
**

**Supplemental Figure 2** – Deletion of alpha-Parvin in skeletal muscle does not alter the protein content of ILK or PINCH. Immunoblotting was performed on gastrocnemius lysates from mice with skeletal muscle specific deletion of alpha-Parvin versus littermate controls. Independent samples T tests were conducted to test differences between groups. Data are presented as mean ± SE. n=4/genotype.

**
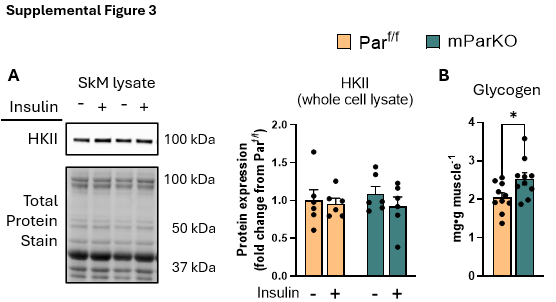
**

**Supplemental Figure 3 – Skeletal muscle expression of HKII and glycogen content.** A) gastrocnemius muscle lysates were generated and prepped for immunoblotting of hexokinase II (HKII). B) Muscle glycogen content following an insulin clamp. Data are presented as mean ± SE. n=6-10/group. Two-way ANOVA with insulin and genotype as factors were run to test statistical differences between groups. T tests were performed in Panel B. *p<0.05

**
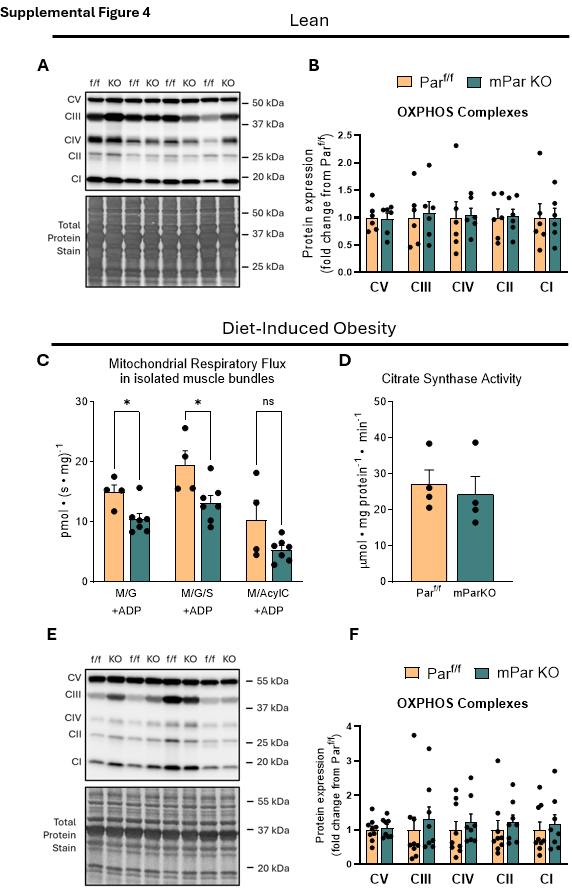
**

**Supplemental Figure 4 – Skeletal muscle mitochondrial complex content and respiratory activity.** A&B) Mitochondrial OXPHOS complexes were probed via immunoblot in lean f/f and mParKO mice. C) Skeletal muscle respiratory flux is decreased in diet-induced obese mParKO mice. Gastrocnemius muscle bundles were isolated and incubated in substrate supplemented media to quantify muscle oxygen consumption. Malate (M) and Glutamate (G) were used to assess complex I mediated respiration; M, G, and Succinate (S) cocktail determines complex II consumption; and M plus Acyl-CoA assesses fatty acid fueled oxygen consumption. D) Citrate synthase activity was measured in gastrocnemius muscle as a proxy for mitochondrial content. E&F) Mitochondrial complexes were measured via immunoblot in DIO f/f and mParKO mice. In Panel C, all substrate conditions were measured in the presence of 2mM ADP. T tests were run to test for differences between groups. Data are presented as mean ± SE. n=6-9/genotype for immunoblotting and n=4-7/genotype for respiratory activity and citrate synthase activity. T tests were performed to determine statistically significant differences between groups. *p<0.05

**
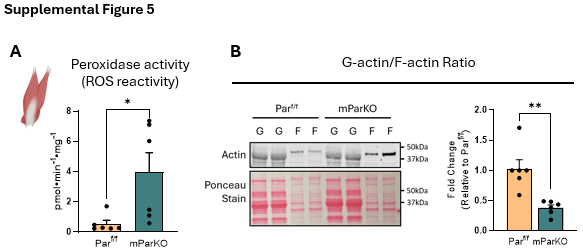
**

**Supplemental Figure 5 - ROS reactivity and G/F actin ratio in skeletal muscle.** A) Skeletal muscle lysates were prepared from Par^f/f^ vs mParKO mice. Peroxidase activity assay is a readout of ROS reactivity. mParKO mice had significantly elevated muscle ROS compared with f/f controls. B) Representative Western blot showing G-actin and F-actin levels in Par^f/f^ and mParKO muscles. Densitometric analysis shows a significant decrease in the G-to-F-actin ratio in mParKO muscle. G, G-actin; F, F-actin. Data are presented as mean ± SE. Student t tests were run to determine significance at alpha <0.05. n=6/genotype. **p<0.01
